# Supramolecular Interaction of Atenolol and Propranolol with β-Cyclodextrin Spectroscopic Characterization and Analytical Application

**DOI:** 10.3390/molecules29122875

**Published:** 2024-06-17

**Authors:** Hebah Alramadhan, Abdalla Ahmed Elbashir, Ahmed O. Alnajjar

**Affiliations:** 1 Department of Chemistry, College of Science, King Faisal University, Al-Hofuf 31982, Al-Ahsa, Saudi Arabia; 218007869@student.kfu.edu.sa (H.A.); anajjar@kfu.edu.sa (A.O.A.); 2 Department of Chemistry, Faculty of Science, Khartoum University, P.O. Box 321, Khartoum 11114, Sudan

**Keywords:** atenolol, propranolol, β-cyclodextrin, inclusion complex

## Abstract

Atenolol (ATE) and propranolol (PRO) inclusion complexes with β-cyclodextrin have been investigated in aqueous solution. The aqueous solution was examined and characterized using UV–vis, fluorescence spectroscopy, and ^1^H NMR. The physical mixture was characterized using FTIR. The existence of inclusion complexes is confirmed by observing changes in spectroscopic properties. The ATE complex with β-CD exhibited an interaction as host and (β-CD) as a guest in a 1:1 ratio, with an inclusion constant K of 2.09 × 10^−3^ µM^−1^, as determined by the typical double-reciprocal graphs. Similarly, the PRO complex with β-CD exhibited an interaction as host and (β-CD) guest in 1:1 and 1:2 stoichiometry at the same time; the inclusion constants were K1 = 5.80 × 10^−5^ µM^−1^ and K2 = 4.67 × 10^−8^ µM^−1^, as determined by typical double-reciprocal graphs. The variables influencing the formation of the inclusion complexes were investigated and optimized. Based on the enhancement in fluorescence intensity due to the formation of inclusion complexes, spectrofluorometric methods were developed and validated for determination of each drug’s pharmaceutical formulation. The quantification of the fluorescence intensity for ATE and PRO was conducted at λ_ex_/λ_em_ 226/302 nm and λ_ex_/λ_em_ 231/338 nm, respectively. Under the optimal reaction circumstances, linear relationships with good correlation coefficients of 0.9918 and 0.99 were found in the concentration ranges of 0.3–1.7 μM, and 0.1–1.1 μM for ATE and PRO, respectively. The limits of detection (LODs) were found to be 0.13 and 0.01 μM for ATE and PRO, respectively. The suggested approach was effectively applied to the analysis of both drugs’ pharmaceutical formulations.

## 1. Introduction

**Figure 1:**
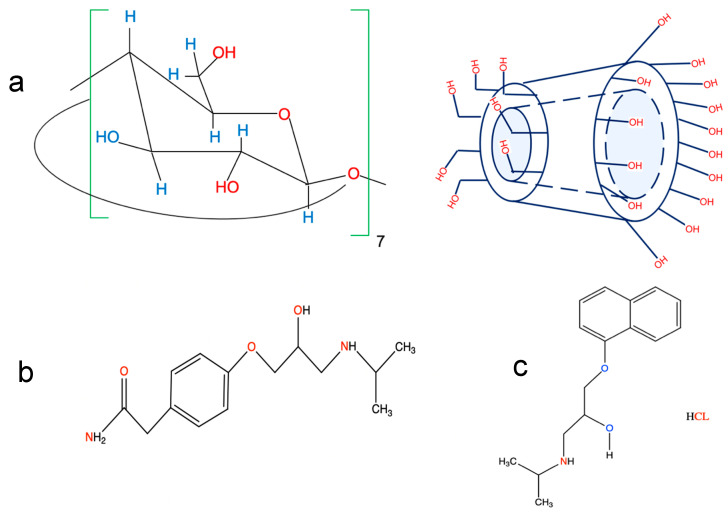
Chemical structures of (**a**) β-CD, (**b**) atenolol (ATE), and (**c**) propranolol (PRO).

Cyclodextrins (CDs) are chiral oligosaccharides that have a cyclic, truncated cone form and are made up of glucopyranose units linked together by (α-1-4) glucoside bonds [1]. The inside of the truncated cone is typically hydrophobic in nature, and thus, effectively traps many chemicals, particularly medications, whereas the external boundary of the truncated cone exhibits many hydroxyl groups, which contribute to its hydrophilic nature (Figure 1a) [2]. The formation of an inclusion complex has a substantial influence on the physicochemical characteristics of the guest molecule, including its solubility, spectroscopic properties, and electrochemical properties. These properties have been used in the pharmaceutical industry precisely to enhance the bioavailability and solubility of medication, by acting as conveyors for chemical compounds in biological cells and organisms [3,4]. From an analytical perspective, the incorporation of compounds enhances the emission intensity [5,6,7,8,9], and induces the separation of chiral compounds in capillary electrophoresis (CE) [10,11]. Moreover, in pharmaceutical and environmental samples, the fluorescent behavior of trapped drugs with CDs was successfully used to create several methods for identifying different chemicals [12,13].

β-blockers are commonly given as cardiovascular medications. These medications are utilized in the treatment of cardiovascular conditions, angina pectoris, including hypertension, myocardial infarction, and cardiac arrhythmias [14]. Atenolol (ATE) 4-(2-hydroxy-3-isopropyl-aminopropoxy) phenylacetamide, is a cardio-selective β-blocker. It can be used independently or in combination with other antihypertensive drugs, such as hydralazine, prazosin, α-methyldopa, and thiazide-type diuretics (Figure 1b) [15]. Moreover, propranolol HCL–(PRO), (RS)-2-(4-(2-methylpropyl)phenyl)-2-propanol is the drug that has been used the most in clinical settings and indications. It is used to treat tremors, angina, hypertension, and heart rhythm disorders (Figure 1c) [16].

β-blockers have poor solubility in aqueous medium; therefore, the low solubility rate and the variability of their bioavailability directly affect the efficiency of the drug. Therefore, with the purpose of increasing the solubility, ATE and PRO were complexed with β-CD and with 2-hydroxypropyl-β-CD to form inclusion complexes [17,18]. A literature survey reveals that some methods have been used to determine ATE and PRO individually in pharmaceutical formulations and biological fluids like capillary electrophoresis [19,20], spectrophotometric methods [21,22], high-performance liquid chromatography [15,23], spectrofluorometric methods [24,25], and gas chromatography [26,27]. 

The inclusion complexes of ATE and PRO with β-CD were studied in solution and investigated using various spectroscopic techniques such as UV–vis spectroscopy, fluorescence spectroscopy, and ^1^H NMR; further, Fourier transform infrared spectroscopy (FTIR) was used for investigation of physical mixtures. To the best of our knowledge spectrofluorometric methods based on inclusion complex formation of ATE and PRO with β-CD have not been reported. Therefore, this work is devoted to the validation and development of spectrofluorometric methods for the determination of ATE and PRO in pharmaceutical formulation. 

## 2. Results and Discussion

### 2.1. Absorption Spectral Characterizations 

**Figure 2:**
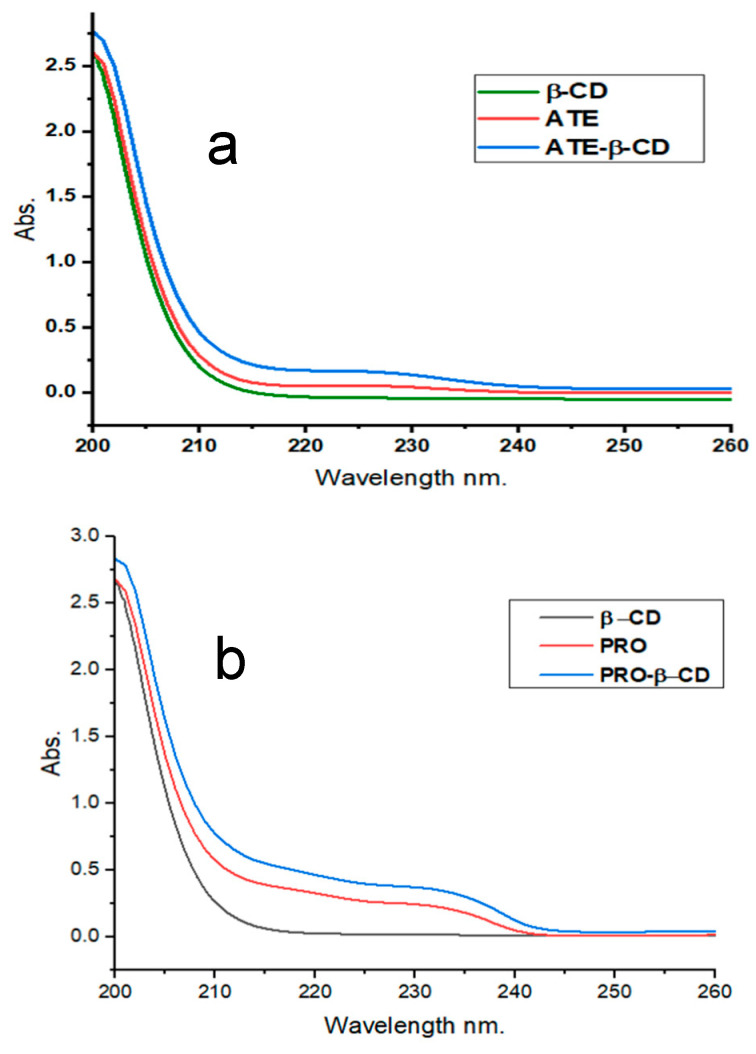
Absorbance spectra for (**a**) ATE-β-CD, 10 μM ATE, 6 × 10^3^ μM β-CD, for time 10 min, at room temperature, pH 7.0; and (**b**) 10 μM PRO, 6 × 10^3^ μM β-CD, for time 10 min, at room temperature, pH 7.0.

The absorption spectra of ATE and PRO were measured without and with the addition of 6 × 10^3^ μM β-CD, and the results are presented in Figure 2a and Figure 2b, respectively. The obtained results indicate that the wavelengths at which ATE and PRO exhibited the highest absorption wavelengths at pH 7.0 were 226 nm and 231 nm. Following the incorporation of β-CD in the solution, the wavelength of maximum absorbance remained constant. However, there was a slight increase in absorbance, leading to an increase in molar absorptivity coefficients ε (L·mol^−1^·cm^−1^) from 22,800 to 36,500 and 5800 to 16,300, for ATE and PRO, respectively. 

### 2.2. Analysis of Infrared Spectra

**Figure 3:**
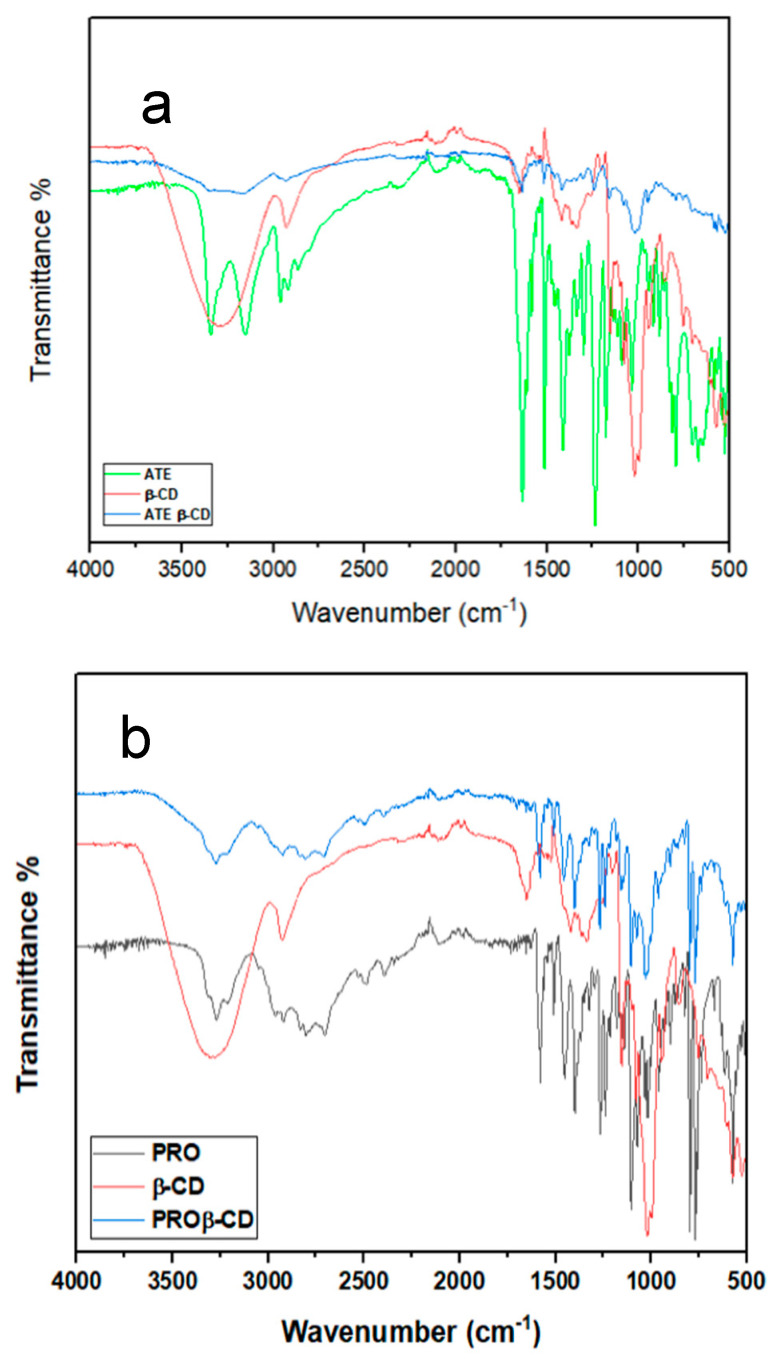
FTIR spectra of (**a**) ATE, β-CD, and ATE-β-CD (**b**) PRO, β-CD, and PRO-β-CD.

In the infrared spectrum of ATE (Figure 3a), the bands were identified as follows: 3342 cm^−1^ for symmetric and asymmetric stretching N-H, 2961 cm^−1^ for alcoholic OH, 1631 cm^−1^ for C=O amide, and 1235 cm^−1^ for alkyl aryl ether. The aforementioned observations match with the FTIR spectra relating to ATE as documented in reference [21]. A number of changes in the FTIR spectra were observed after the formation of the ATE-β-CD inclusion complex. The band assigned to the N-H stretching disappeared. The band assigned to the OH group was weaker and shifted towards shorter wavelengths, to 2910 cm^−1^. The C=O amide band at 1636 cm^−1^ was shifted towards shorter wavelengths, to 1635 cm^−1^, and was weaker. Furthermore, the O-H in the β-CD band at 3391 cm^−1^ was shifted towards shorter wavelengths, to 3199 cm^−1^, and was weaker in the inclusion complex than in the original β-CD spectrum. The IR spectrum of the PRO (Figure 3b) includes several bands; first, at 3270 cm^−1^ belonging to the secondary OH group, 2919 cm^−1^ to N-H stretching, 1104 cm^−1^ for the aryl alkyl ether; also, the band at 768 cm^−1^ belongs to *α*-substituted naphthalene. The aforementioned observations match with the FTIR spectra relating to PRO as documented in reference [28]. Obviously, there were significant alterations in the FTIR spectra after the complex PRO-β-CD formed. The secondary OH group band at 3299 cm^−1^ was shifted to approximately 3300 cm^−1^. The band at 2919 cm^−1^ for NH stretching was shifted to a shorter wavelength at 2900 cm^−1^. The band belonging to the aryl alkyl ether at 1104 cm^−1^ was shifted to 1019 cm^−1^. Also, the band belonging to the *α*-substituted naphthalene at 768 cm^−1^was shorter, weaker, and shifted to a longer wavelength (blue shift), to 769 cm^−1^. The previously mentioned alterations can possibly be attributed to variations in the microenvironment, which contribute to van der Waals forces, and the existence of hydrogen bonding during the connection between them, which eventually leads to the formation of the complexes of inclusion between ATE and PRO and β-CD in the solid physical mixture. 

### 2.3. ^1^H NMR Spectroscopy

One of the efficient methods for studying CDs complexation is ^1^H NMR spectroscopy [29,30]. The possibility of forming an inclusion complex can possibly be determined by analyzing the ^1^H NMR chemical shift pattern of the cyclodextrin (CD) and the guest molecule. Hydrophobic interactions, van der Waals forces, and hydrogen bonds are examples of non-covalent bonds that connect host and guest molecules. Their chemical shifts will change if the guest molecules ATE and PRO are trapped in the cyclodextrin β-CD cavity. The ^1^H chemical shift values of ATE and PRO, both before incorporation and after the formation of the inclusion complex, are shown in Table 1 and Figures S1 and S2. The values obtained clearly demonstrate that complexation induced significant chemical shifts in the ATE and PRO protons, providing confirmation of the formation of the complexes.

### 2.4. Emission Spectra Characterizations

**Figure 4:**
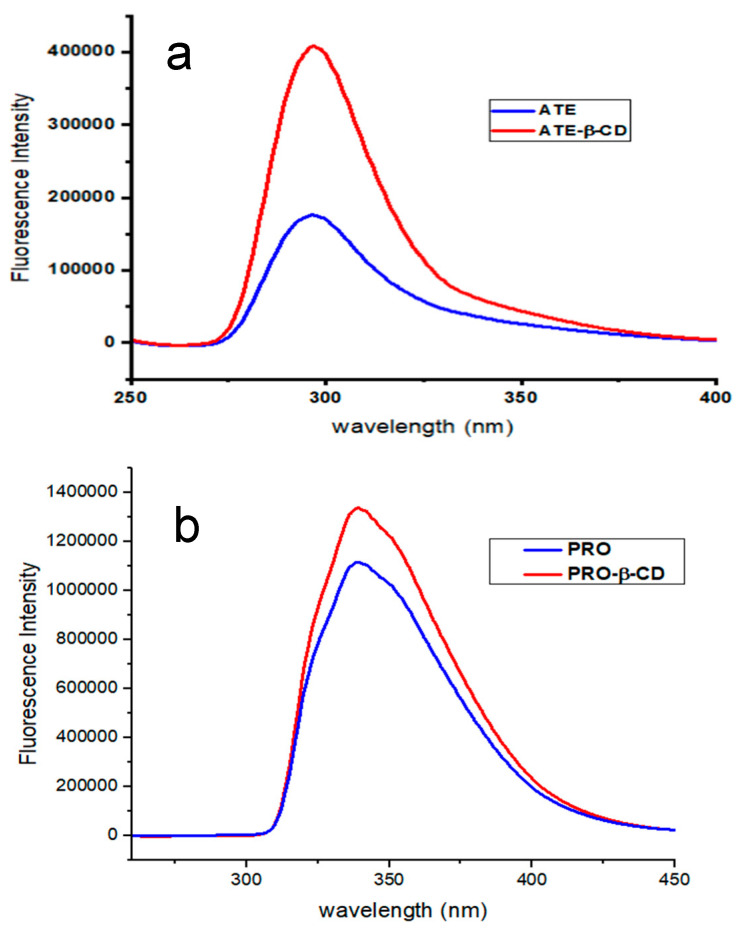
Emission spectra of (**a**) ATE-β-CD, 10 μM ATE, 6 × 10^3^ μM β-CD, in 10 min period, at room temperature, pH 7.0; and (**b**) PRO-β-CD, 10 μM PRO, 6 × 10^3^ μM β-CD, in 10 min period, at room temperature, pH 7.0.

Fluorescence spectroscopy is a highly sensitive and selective tool for studying host–guest molecular systems. Fluorescence analysis has significant advantages in terms of its enhanced selectivity and ability to quantify much lower concentrations when compared to spectrophotometric analysis. Initially, analysis was conducted on the spectral characteristics of ATE and PRO. The study findings indicated that the maximal emission wavelengths of ATE and PRO at pH 7.0 were 297 nm and 338 nm, respectively. When β-CD was added into each drug flask, the fluorescence intensity rose, at the same time, the emission’s maximum wavelength remained consistent, as demonstrated in Figure 4a,b. The explanation of this occurrence may be attributed to the fact that ATE and PRO are introduced into the hydrophobic cavity of β-CD, where they are included by non-covalent bondings such as hydrogen bonding and van der Waals forces.

### 2.5. Optimization of Parameters Affecting Inclusion Complex Formation 

Several factors that affect the inclusion complex formation such as the pH of the buffer, the complexation time, and the concentration of β-CD were studied. 

#### 2.5.1. Optimization of the pH of the Inclusion Complexes 

**Figure 5:**
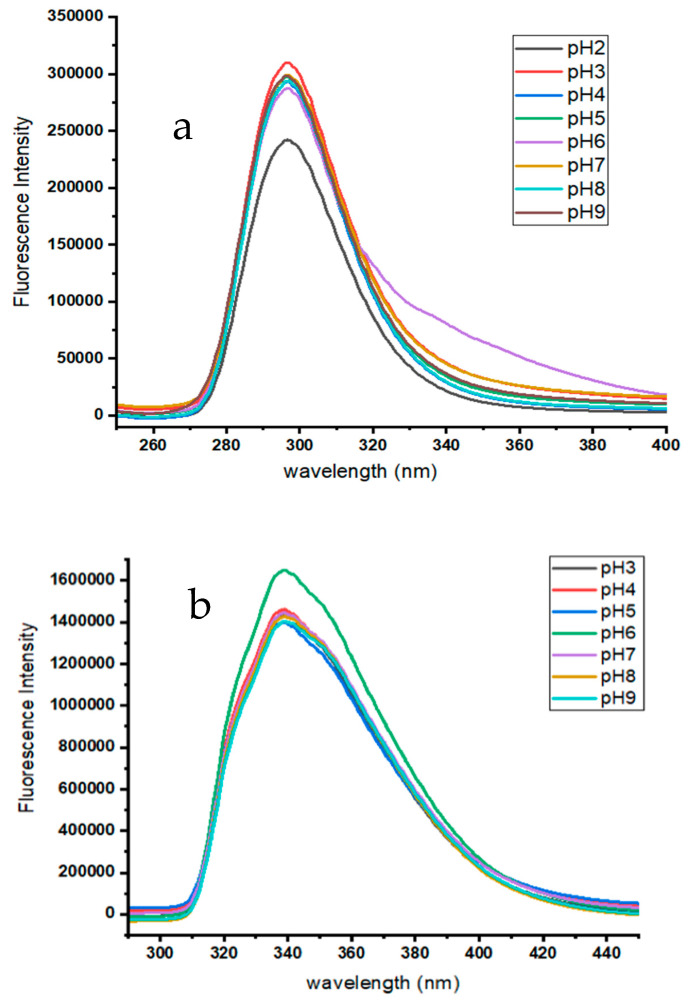
Emission spectra: (**a**) ATE-β-CD in series of pH from pH 2.0 to pH 9.0. 10 μM ATE; 200 μM β-CD; at room temperature; time 10 min; the highest fluorescence intensity was at pH 3.0. (**b**) PRO-β-CD in series of pH from pH 3.0 to pH 9.0. 10 μM PRO; 200 μM β-CD; at room temperature; time 10 min; the highest fluorescence intensity was at pH 6.0.

The pH has a significant impact on the formation of ATE and PRO inclusion complexes with β-CD. The pH was examined in the range from 2 to 9, and the results are presented in Figure 5a,b. The results indicate that the fluorescence intensity initially remained constant, then rose sharply at pH 3.0 for ATE and at pH 6.0 for PRO; after that, it dropped back to its original values. The optimum pH values for the inclusion complexes are therefore determined to be 3.0 and 6.0, respectively. The pKa value for ATE is reportedly 9.6 and for PRO 9.4 [31]. This confirms that the protonated form is the most suitable for the inclusion complexes. 

#### 2.5.2. Optimization of the Complexation Time

The effect of time on the fluorescence intensity of the complexes formation of ATE and PRO with β-CD was studied. The experiment was conducted at room temperature; in range from 5 to 25 min; the results obtained indicate that the highest fluorescence intensity readings were obtained at 10 and 20 min for ATE and PRO, respectively. 

#### 2.5.3. Optimization of β-CD Concentration 

**Figure 6:**
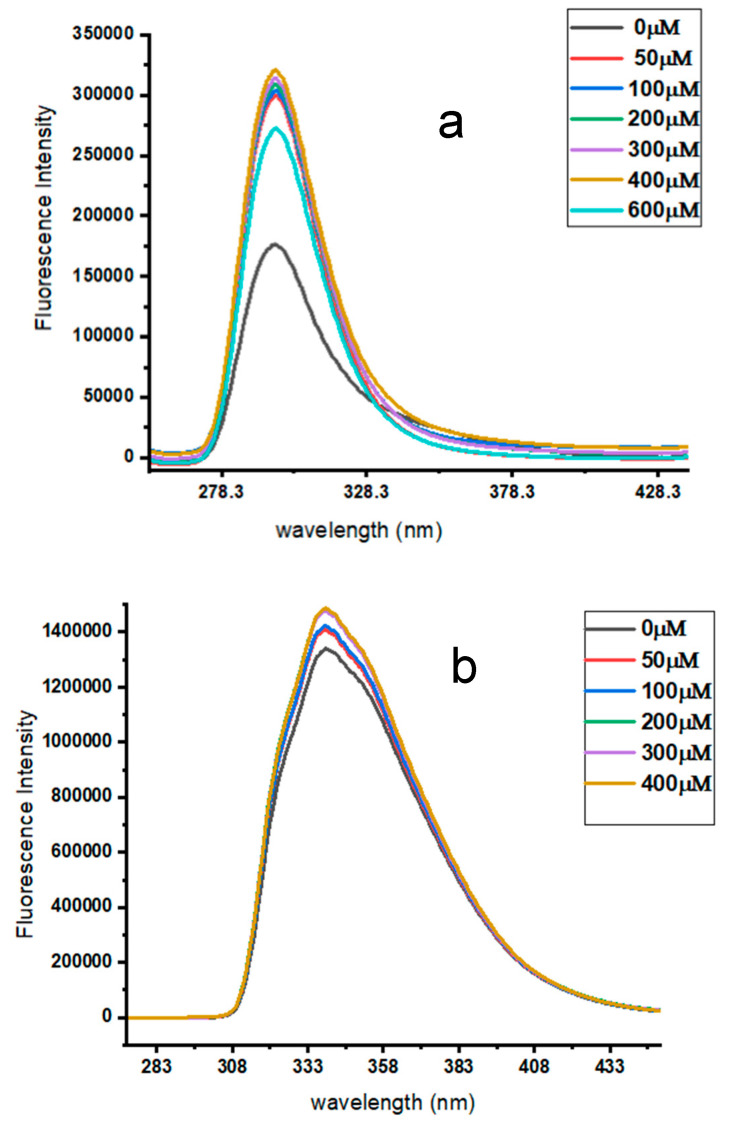
Emission spectra of (**a**) 10 μM ATE with different concentrations of β-CD, from 50 μM to 600 μM, for a time of 10 min, pH 3.0, at room temperature; and (**b**) 10 μM PRO with different concentrations of β-CD, from 50 to 400 μM, in a time of 10 min, pH 6.0, at room temperature.

In order to optimize the β-CD concentration, the drug’s concentration was kept constant at 10 μM, whereas the β-CD concentration was varied between 50 and 600 μM, and 50 and 400 μM for ATE and PRO, respectively. The fluorescence intensity increased steadily until it reached a stable inclusion complex at 400 and 200 μM for ATE and PRO, respectively. Above this point the fluorescence intensity remained constant, as shown in Figure 6a,b. Therefore, these values were used as optimum values. 

### 2.6. Stoichiometry of Inclusion Complexes

**Figure 7:**
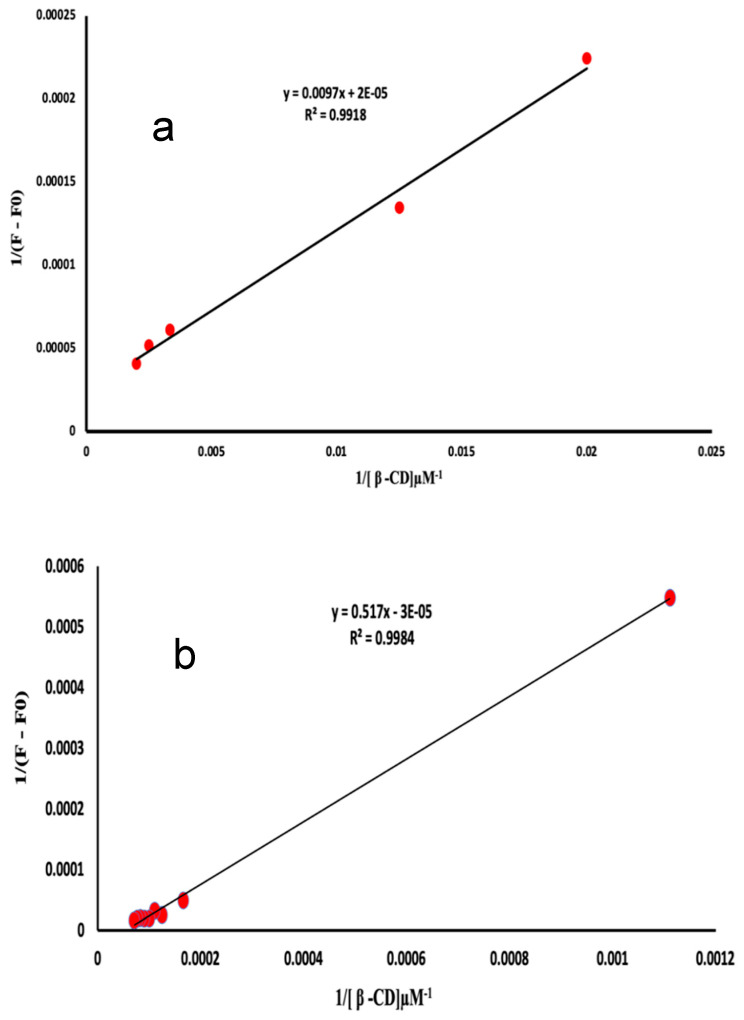
Plot of (**a**) 1/(F − F0) vs. 1/[β-CD] of ATE-β-CD complex, 10 μM ATE, pH 3.0; and (**b**) plot of 1/(F − F0) vs. 1/[β-CD] of PRO-β-CD complex, 10 μM PRO, pH 6.0.

**Figure 8:**
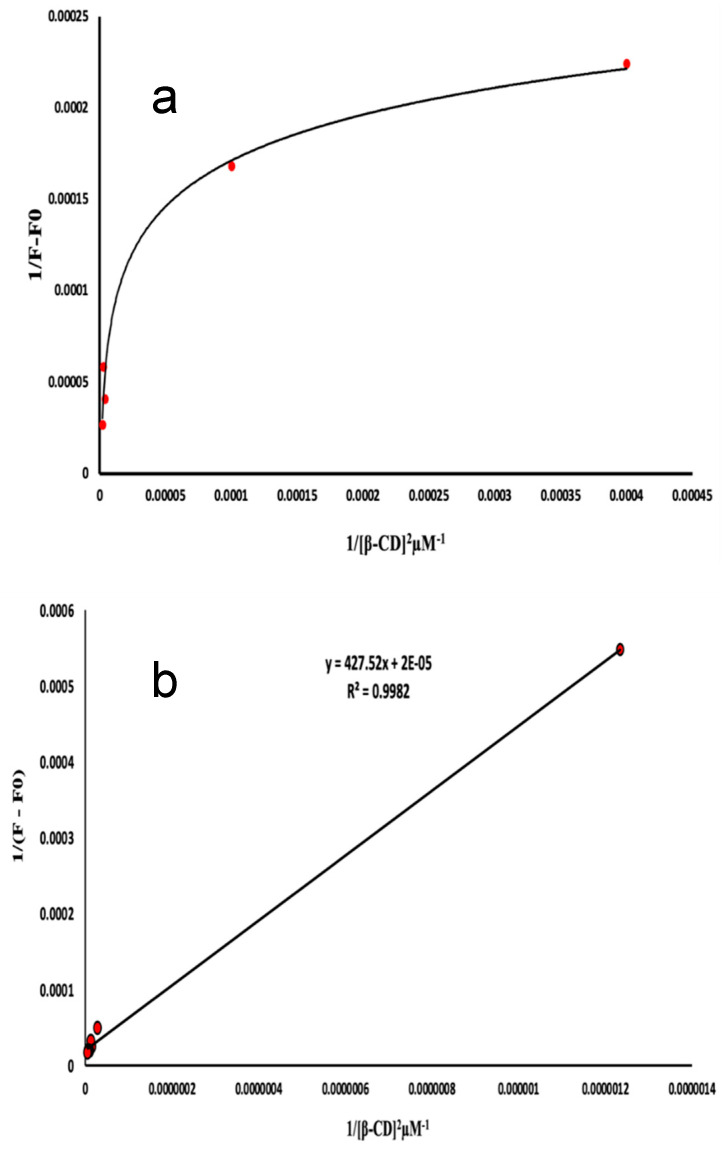
Plot of (**a**) 1/(F − F0) vs. 1/[β-CD]^2^ of PRO-β-CD complex, 10 μM PRO, pH 6.0; and (**b**) plot of 1/(F − F0) vs. 1/[β-CD]^2^ of ATE-β-CD complex, 10 μM ATE, pH 3.0.

The stoichiometry of the inclusion complexes were analyzed using the experimental parameters that had been optimized earlier. It was assumed that the complexes had a 1:1 ratio. The study included an analytical technique, namely, Benesi–Hildebrand plots. The equation is formulated as follows: **1/F − F0 = 1/(F∞ − F0)K[β-CD**0**] + 1/F∞ − F0**. In this equation, F∞ represents the fluorescence intensity when the majority of drug molecules are trapped in β-CD, [β-CD0] represents the initial concentration of β-CD, and F represents the observed fluorescence intensity at every measured concentration of β-CD. Plots of 1/F − F0 vs. 1/[β-CD0] (Figure 7a,b) suggest a 1:1 stoichiometry for the ATE-β-CD and PRO-β-CD complexes, with inclusion constants of K = 2.09 × 10^−3^ µM^−1^ and K = 5.80 × 10^−05^ µM^−1^, respectively. In order to precisely evaluate the value of c, the determination of the stoichiometric ratio, assuming 1:2 inclusion complexes with β-CD, a plot of **1/F** − **F0 = 1/(F∞** − **F0)K2([β-CD**0**]**)^2^**+1/F∞** − **F0** is used. By plotting 1/(F − F0) as a function of 1/[β-CD]^2^ (Figure 8a) a linear correlation was obtained that confirmed that there is a possibility to form both 1:1 and 1:2 inclusion complexes of PRO with β-CD at the same time. However, by plotting 1/(F − F0) as a function of 1/[β-CD]^2^ for ATE-β-CD (Figure 8b), the non-linearity can be observed, from which it can be concluded that the stoichiometry of the complex is 1:1 [32]. 

### 2.7. Validation of the Analytical Methods

The analytical procedures were validated in terms of linearity, and limits of detection (LODs) and quantification (LOQs), precision, accuracy, and robustness in accordance with international conference harmonization (ICH) [33]. 

#### 2.7.1. Linearity, LODs, and LOQs

The suggested procedures showed a linear relationship for ATE-β-CD with a good correlation coefficient (*n* = 8) for concentrations ranging from 0.3 to 1.7 μM. A linear plot was generated by PRO-β-CD with n = 6, and showed a good correlation coefficient within the concentration range of 0.1–1.1 μM. Table 2 shows the quantitative parameters and statistical data. The LODs and LOQs were found to be 0.13 and 0.40 μM, and 0.01 and 0.3 μM, for ATE and PRO, respectively. 

#### 2.7.2. Accuracy and Precision of Spectrofluorometric Methods 

The accuracy and precision of the suggested procedures were assessed by examining three triplicate samples at three different concentration levels of ATE and PRO. The results obtained exhibit a significant level of consistently and accuracy (Table 3), indicating the reliability of the procedure. The high precision was ideal to use for assurance of the quality of ATE and PRO in their pharmaceutical formulation. 

#### 2.7.3. Robustness of Spectrofluorometric Methods 

Robustness was assessed by analyzing how slight parameter changes affected the analytical results. In these experiments, the recovery was calculated on each case as a single parameter was modified while keeping the others constant. Consequently, it was found that minor changes in the technique parameters did not have a major effect on the operations. The recovery values are shown in Table 4. 

#### 2.7.4. Analysis of Pharmaceutical Formulations 

The proposed methods were successfully used for analysis of pharmaceutical formulations of ATE and PRO with acceptable accuracy. The percentage values for ATE and PRO were 103% and 100%, respectively, based on five determinations, as presented in Table 5. 

## 3. Experimental

### 3.1. Chemical Reagents

β-CD, monobasic and dibasic sodium phosphate, ATE, and PRO standards were purchased from Sigma-Aldrich (St. Louis, MO, USA).

### 3.2. Instruments and Apparatus 

The Shimadzu RF 6000 spectrofluorometer was used for conducting fluorescence spectra and intensity measurements, manufactured by Shimadzu in Japan (Kyoto, Japan), which included a 150 W xenon lamp. The spectrophotometric measurements were performed using a Shimadzu UV-1650 pc double-beam ultraviolet–visible spectrophotometer from Japan, which included a 50 W halogen lamp and a deuterium lamp. Also, IR was recorded using a spectrometer (Cary 630 FTIR). ^1^H NMR spectra were run using a Bruker Ascend 400 spectrometer (Bruker, Billerica, MA, USA) with the TopSpin 3.5 software.

### 3.3. Preparation of Standard Solutions of Drugs and Cyclodextrin 

A volume of 1000 μM of ATE stock solution was made by dissolving 6.6 mg of ATE in phosphate buffer at pH 3.0; similarly, 1000 μM of PRO stock solution was generated by dissolving 7.4 mg of PRO in phosphate buffer at pH 6.0; both solutions were placed into a 25 mL standard flask and phosphate buffer with the same pH was added to them in order to achieve dilution. In order to prepare a β-CD stock solution of 15,000 μM, 1.7 g was submerged in deionized water then poured into a 100 mL standard flask, and then, more deionized water was added in order to achieve dilution. 

### 3.4. Buffer Solutions 

Buffer solutions with pH values of 6.0 and 7.0 were prepared by utilizing 0.1 M NaH_2_PO_4_ and 0.1 M Na_2_HPO_4_. Additionally, 0.1 M H_3_PO_4_ and 0.1 M NaH_2_PO_4_ were used to prepare a buffer solution with a pH of 3.0, which was adjusted using a pH meter. 

### 3.5. UV–Visible Spectroscopy Measurement

Into separate 10 mL volumetric flasks, 0.1 mL of each drug solution was placed. Subsequently, a volume of 4 mL of the solution of β-CD was transferred to each flask. The remaining volume in each flask was then filled with phosphate buffer at pH 7.0. The final mixed solutions were exposed to oscillation at room temperature by putting them in a sonicator bath for a duration of 10 min. Next, the absorbance spectra were determined at wavelengths of 226 nm and 231 nm, for ATE and PRO, respectively. 

### 3.6. Fluorescence Measurement

A volume of 0.1 mL of ATE (1000 μM) and 4 mL of β-CD were placed into a 10 mL volumetric flask. The final mixture was completed and diluted to the desired volume using a buffer solution of phosphate of pH 3.0 to obtain the final concentration of 10 μM. The flask was put in the sonicator at ambient temperature for 10 min. The fluorescence intensity of the complex formed was measured at λ_ex_/λ_em_ 226/297 nm. Similarly, PRO-β-CD was prepared with 0.1 mL of PRO (1000 μM) and 4 mL of β-CD added into a 10 mL volumetric flask to obtain the final concentration of 10 μM. The final mixture was completed and diluted to the desired concentration with phosphate buffer at pH 6.0, after that, the flask was put in the sonicator at ambient temperature for 10 min. The intensity of fluorescence of the inclusion complex formed by PRO and β-CD was analyzed at λ_ex_/λ_em_ 231/338 nm.

### 3.7. Determination of Stochiometric Ratio

The inclusion complexes’ stochiometric ratios were investigated using Benesi–Hildebrand plots. The equation is formulated as follows: 1/F − F0 = 1/(F∞ − F0)K[β-CD0] + 1/F∞ − F0. In this equation, F∞ represents the fluorescence intensity when the majority of drug molecules are trapped in β-CD, [β-CD0] represents the initial concentration of β-CD, and F represents the observed fluorescence intensity at every measured β-CD concentration.

### 3.8. Limits of Detection (LODs) and Limits of Quantification (LODs) 

The LODs and LOQs were calculated using the following equation: LOD or LOQ = K. SDa/b, where the symbol K denotes the value 3.3 in the case of calculating LOD, and in the case of calculating LOQ is 10. The symbol SDa refers to the standard deviation of the intercept, whereas b represents the slope. 

## 4. Conclusions

This research provided evidence that β-CDs have the ability to function as guest-complexing agents. The use of UV–visible spectroscopy, fluorescence spectroscopy, ^1^H NMR, and FTIR demonstrated the formation of inclusion complexes between ATE and PRO with β-CD. The methods are designed to accurately detect each drug when β-CD is present by increasing fluorescence intensity during complexation formation. The methods are simple and sensitive, comprehensively validated, and successfully employed for analysis of the two drugs in their pharmaceutical formulations.

## Figures and Tables

**Table 1:** NMR of ATE and PRO and the shift after complexation with β-CD.

Proton ATE	ATE Free (ppm) ^δ^	ATE Complexed (ppm) ^δ^	∆ ^δ^ (ATE Complexed-ATE Free) (ppm)
2H-2′H	7.144	7.0870	−0.057
3H-3′H	6.892	6.820	−0.072
6H-6′H	4.00	4.867	0.867
5H	3.905	3.631	−0.27
1H-1′H	3.439	4.821	1.3
4H-4′H	2.739	2.740	0.001
7H	2.597	2.592	−0.005
8H-8′H	0.944	0.945	0.001
**∆ ^δ^ (PRO Complexed—PRO Free) (ppm)**	**PRO Complexed (ppm) ^δ^**	**PRO Free (ppm) ^δ^**	**Proton PRO**
14-H	8.219	8.099	0.12
7-H	7.662	7.776	−0.114
4-H	7.643	7.769	−0.126
3-H,5-H,6-H	7.467	7.471	−0.004
2-H	7.327	7.430	−0.103
1-H	6.001	6.819	−0.818
9-H	4.911	4.694	0.217
8-H	4.258	4.111	0.147
8′-H	4.260	4.131	0.129
12-H	3.916	3.525	0.391
10′-H	3.287	3.206	0.081
10-H	3.186	3.106	0.08
11-H,13-H	1.194	1.268	−0.074

^δ^ Means the change.

**Table 2:** Parameters for ATE-β-CD and PRO-β-CD spectrofluorimetric methods.

Parameter	Value (ATE)	Value (PRO)
Linear range	0.3–1.7 μM	0.1–1.1 μM
Slope	31,015	152,649
Intercept	431.73	15,307
LOD (μM)	0.13	0.01
LOQ (μM)	0.40	0.3
Correlation coefficient (r)	0.99	0.99
Optimum pH	pH = 3.0	pH = 6
Optimum β-CD concentration	400 μM	200 μM
Optimum complexation time	10 min	20 min

**Table 3:** The accuracy and precision of ATE-β-CD and PRO-β-CD.

Sample Content (μM)	Standard Added (μM)	Amount Found (μM)	Recovery% ± RSD *
ATE			
0.5	0.1	0.61	101% ± 1.8
0.5	0.4	0.85	94.4% ± 1.0
0.5	0.6	1.12	101% ± 0.2
**PRO**			
0.2	0.1	0.38	126% ± 2.2
0.2	0.4	0.7	116% ± 3.3
0.2	0.9	1.17	106% ± 5.5

* Values are the mean of three determinations.

**Table 4:** Robustness of the spectrofluorometric method for ATE-β-CD and for PRO-β-CD.

Parameter Standard Condition	Recovery ± SD * 101% ± 0.1
ATE	
pH	
3.2	111% ± 0.3
2.8	122% ± 0.2
β-CD concentration (μM)	
420	107% ± 0.3
380	111% ± 0.2
Reaction time (min)	
10	122% ± 0.2
15	122% ± 0.19
**Parameter** **Standard Condition**	**Recovery** ± SD * 116% ± **0.5**
**PRO**	
pH	
6.2	93.3% ± 3.7
5.8	91.6% ± 0.2
β-CD concentration (μM)	
220	98.3% ± 1.8
180	105% ± 1.2
Reaction time (min)	
20	86.6% ± 2.1
25	91.6% ± 2.0

* Values are mean of three determinations.

**Table 5:** The method applied for analysis of ATE and PRO in pharmaceutical formulations.

Concentration Taken (μM)	Concentration Found (μM)	Recovery% ± RSD *
ATE		
1	1.03	103% ± 0.5
**PRO**		
0.5	0.5	100% ± 4.0

* Values are the means of five determinations.

## Data Availability

The data presented in this study are available in article and Supplementary Materials.

## Supplementary Materials

The following supporting information can be downloaded at: https://www.mdpi.com/article/10.3390/molecules29122875/s1, Figure S1. ^1^H-NMR spectra for (a) β-CD, (b) free ATE (c) ATE-β-CD complex in D_2_O. The molar ratio of βCD: guest is 1:1. Figure S2. ^1^H-NMR spectra for (a) β-CD, (b) free PRO (c) PRO-β-CD complex in D_2_O. The molar ratio of β-CD: guest is 1:2.
