# Supplementary material for: Supramolecular Interaction of Atenolol and Propranolol with β-Cyclodextrin Spectroscopic Characterization and Analytical Application
Source: Molecules. 2024 Jun 17;29(12):2875. doi: 10.3390/molecules29122875 (PMC11206265; doi:10.3390/molecules29122875)
Supplement: Supplementary file 1 [file molecules-29-02875-s001.zip › molecules-3050695-supplementary.pdf]

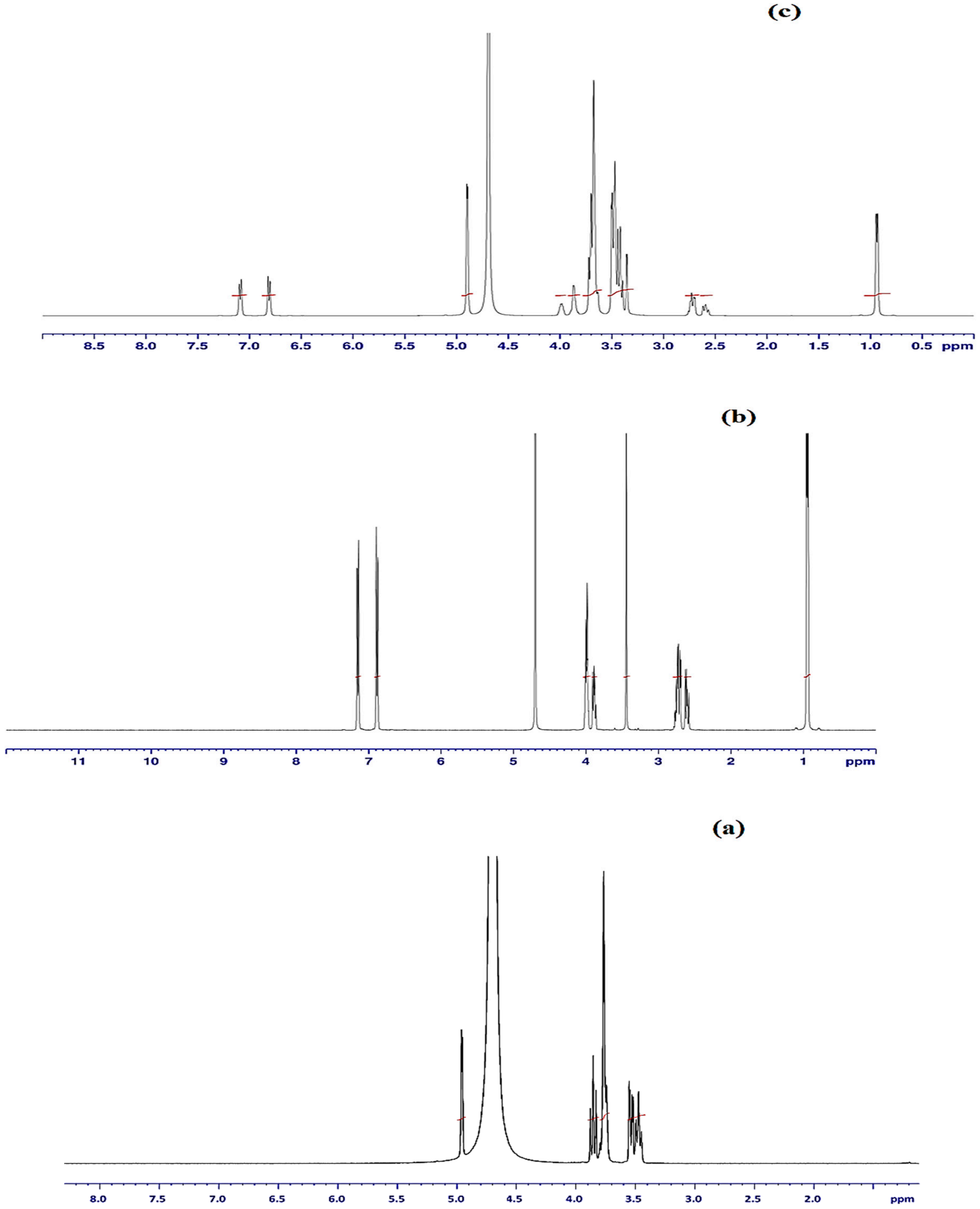

Figure S1 ^{1}H-NMR spectra for (a) β-CD, (b) free ATE (C) ATE-β-CD complex in D_2O. The molar ratio of βCD: guest is 1:1.

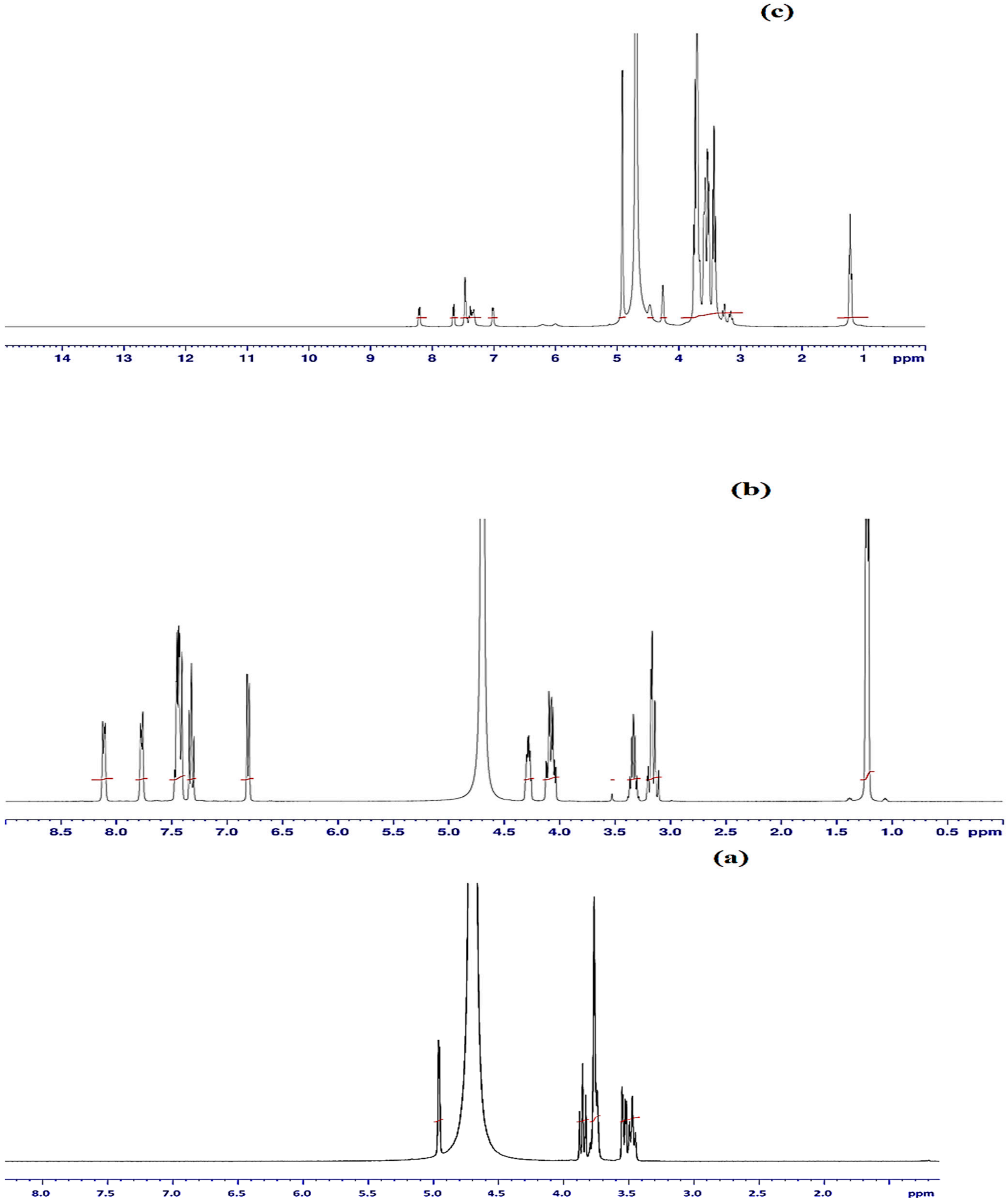

Figure S2 ^{1}H-NMR spectra for (a) β-CD, (b) free PRO (C) PRO-β-CD complex in D_2O. The molar ratio of β-CD: guest is 1:2.
